# Risk-adapted nodal evaluation in stage IA non–small cell lung cancer: a population-based competing-risk analysis

**DOI:** 10.1186/s12957-026-04485-2

**Published:** 2026-07-01

**Authors:** Ziqiang Wang, Yangyang Xie, Tingting Wang, Xue Song, Nian Liu

**Affiliations:** 1 The First People’s Hospital of Xiaoshan District, Xiaoshan Affiliated Hospital of Wenzhou Medical University, Hangzhou, 311000 China; 2 Zhejiang Key Laboratory of Multi-omics Precision Diagnosis and Treatment of Liver Diseases, Department of General Surgery, Sir Run-Run Shaw Hospital, Zhejiang University School of Medicine, Hangzhou, 310016 China; 3 The Department of General Practice, The Second Affiliated Hospital and Yuying Children’s Hospital of Wenzhou Medical University, Wenzhou, 325027 China; 4 Department of Respiratory and Critical Care Medicine, Hangzhou TCM Hospital, Zhejiang Chinese Medical University, Hangzhou, 310007 China

**Keywords:** Early-stage non–small cell lung cancer, Stage IA, Lymph node evaluation, Examined lymph nodes, Competing risk analysis, Propensity score matching

## Abstract

**Background:**

The oncologic value of intensified lymph node (LN) evaluation in stage IA non–small cell lung cancer (NSCLC) remains uncertain in the era of surgical de-escalation. Although expanded nodal assessment may reduce understaging, whether it is associated with meaningful survival differences in this favorable-risk population is unclear. Clarifying this issue is essential to balance oncologic adequacy and surgical burden.

**Method:**

We conducted a population-based cohort study using the Surveillance, Epidemiology, and End Results database and included patients with surgically treated pathologic stage IA (T1N0M0) NSCLC. The extent of nodal evaluation was quantified by the number of examined lymph nodes. Propensity score matching was used to balance baseline characteristics. Cancer-specific and other-cause death were analyzed using cumulative incidence functions and Fine–Gray competing-risk models. Prespecified subgroup analyses assessed heterogeneity of association, particularly according to tumor size.

**Results:**

We included 24,215 patients, with 13,812 after matching. Intensified nodal evaluation, defined as ≥ 6 examined lymph nodes (ELNs), was associated with lower cancer-specific mortality (5-year cumulative incidence, 12.9% vs. 15.3%; absolute risk reduction [ARR] 2.4%; Gray’s *P* < 0.001). In multivariable Fine–Gray analysis, ELNs ≥ 6 remained independently associated with lower cancer-specific mortality (subdistribution hazard ratio 0.78, 95% CI 0.74–0.83). The association was minimal in tumors ≤ 1 cm but more evident in tumors > 1 cm. Sensitivity analyses using ELNs ≥ 10 yielded similar findings.

**Conclusion:**

The association between intensified nodal evaluation and lower cancer-specific mortality in stage IA NSCLC varies by tumor size. This association was more evident in tumors > 1 cm, supporting further investigation of a size-based, risk-adapted approach to surgical staging in early-stage NSCLC.

**Supplementary Information:**

The online version contains supplementary material available at https://doi.org/10.1186/s12957-026-04485-2.

## Introduction

With the widespread implementation of low-dose computed tomography screening, an increasing proportion of non–small cell lung cancers (NSCLC) are diagnosed at stage IA [1]. This shift toward early detection has accelerated the adoption of parenchymal-sparing approaches, particularly sublobar resection, and has fundamentally changed the balance between oncologic adequacy and surgical de-escalation [2]. In this evolving landscape, lymph node (LN) evaluation remains critical for accurate staging and postoperative management [3]. Although systematic evaluation may reduce occult nodal metastasis, it may also increase surgical complexity and morbidity, especially in patients with small or biologically indolent tumors. These considerations highlight the need to better understand whether intensified nodal evaluation is associated with meaningful differences in oncologic outcomes in early-stage NSCLC [4].

Although more extensive LN evaluation has been associated with improved outcomes in early-stage NSCLC, it remains unclear whether these associations reflect differences related to nodal evaluation itself or residual confounding [5]. Most prior studies have focused on prognostic correlations rather than this question in more homogeneous early-stage populations [6]. Moreover, heterogeneous study populations and reliance on conventional survival analyses may obscure cancer-specific outcomes in this favorable-risk setting [7, 8]. As a result, the clinical significance of intensified nodal evaluation in stage IA NSCLC remains uncertain.

Competing-risk analysis provides a more appropriate framework for outcome assessment in diseases with favorable prognosis and prolonged survival [9]. In stage IA NSCLC, long-term survivors are at substantial risk of non–cancer-related death, which may bias conventional survival estimates and misrepresent the association between intensified nodal evaluation and cancer-specific outcomes. Applying a competing-risk approach in a homogeneous early-stage cohort may therefore provide a more clinically relevant assessment and better inform surgical decision-making [10].

In this context, we conducted a large population-based study using the Surveillance, Epidemiology, and End Results database to evaluate the association between intensified nodal evaluation and oncologic outcomes in surgically treated pathologic stage IA NSCLC [11, 12]. By focusing on a homogeneous T1N0M0 cohort and applying a competing-risk framework with cancer-specific death as the primary endpoint, we aimed to clarify whether intensified nodal evaluation was associated with lower cancer-specific mortality in this favorable-risk population [13]. Furthermore, we sought to identify clinically relevant subgroups in whom this association was more evident, thereby informing a risk-adapted and individualized strategy for nodal staging in contemporary early-stage NSCLC[14, 15].

## Methods

### Data source and study population

This population-based cohort study used data from the National Cancer Institute’s Surveillance, Epidemiology, and End Results (SEER) program. Data were extracted with SEER*Stat from *Incidence – SEER Research Data*,* 17 Registries*,* Nov 2024 Submission (2000–2022)*. SEER contains publicly available, de-identified data; therefore, this study required no additional institutional review board approval or informed consent.

**Fig. 1:**
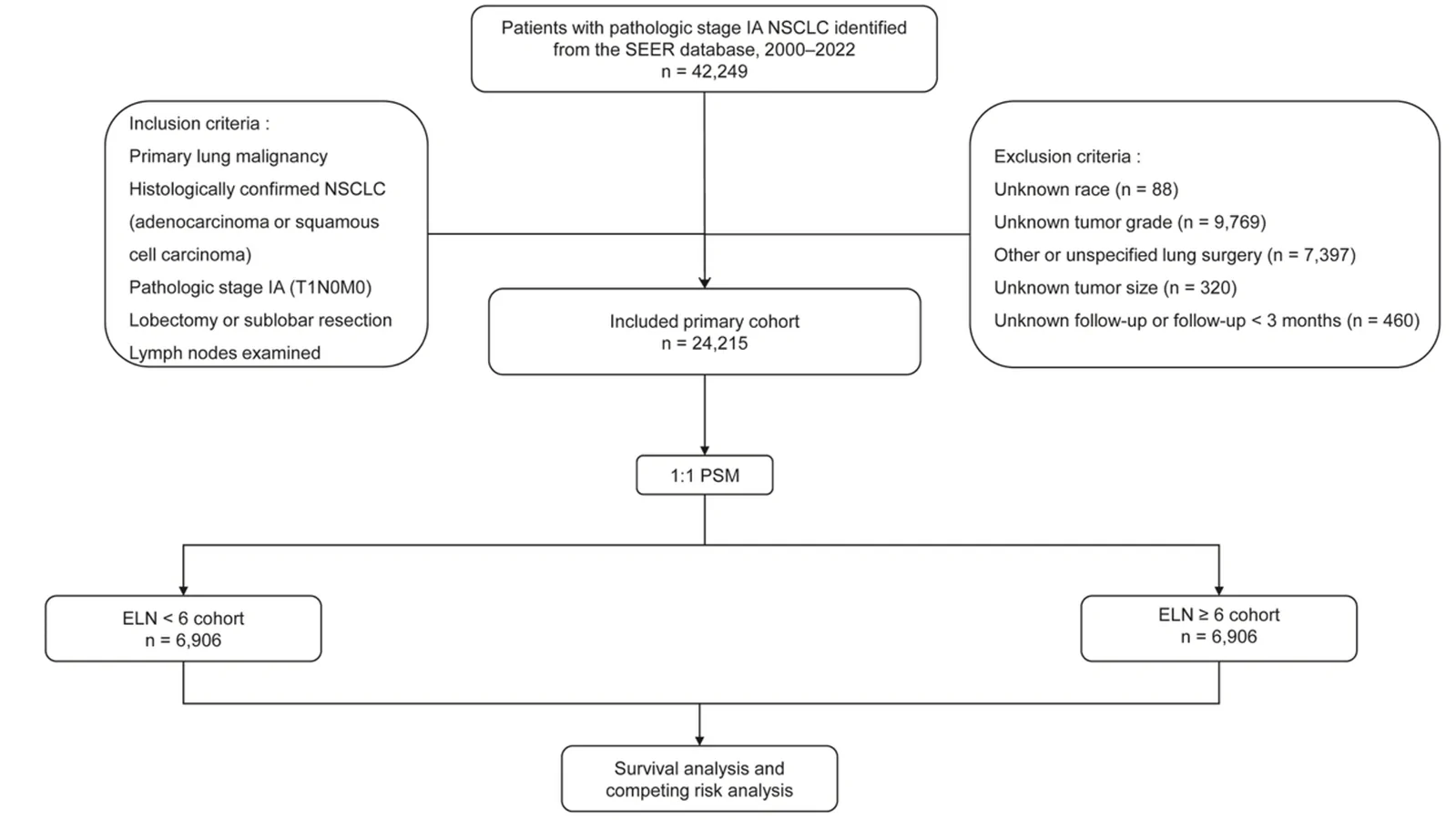
Flowchart of patient selection for the stage IA NSCLC cohort. Abbreviations: NSCLC, non–small cell lung cancer; SEER, Surveillance, Epidemiology, and End Results; ELN, examined lymph nodes; PSM, propensity score matching

We identified patients diagnosed between January 1, 2000 and December 31, 2022 with pathologic stage IA NSCLC, defined as T1N0M0 using the SEER-derived American Joint Committee on Cancer (AJCC) tumor–node–metastasis (TNM) staging variable. During this period, SEER derived TNM categories according to the applicable AJCC 6th–8th editions; however, the core definition of T1 as a tumor ≤ 3 cm with N0M0 status remained consistent across editions. Inclusion criteria were: (1) lung as the primary site and first primary malignancy; (2) histologic confirmation of NSCLC (primarily adenocarcinoma or squamous cell carcinoma); (3) pathologic stage IA (T1N0M0) at diagnosis; (4) receipt of curative pulmonary resection, including lobectomy or sublobar resection (segmentectomy or wedge resection), defined by the “Surgery of primary site” variable; (5) at least one regional lymph node examined (regional nodes examined ≥ 1) with complete recording of the “Regional nodes examined” variable; and (6) survival time ≥ 3 months. Exclusion criteria were missing race (*n* = 88), unknown tumor grade (*n* = 9,769), surgical procedures that could not be categorized as lobectomy or sublobar resection (e.g., no surgery, local tumor destruction, unclear/other complex procedures; *n* = 7,397), unknown tumor size (*n* = 320), and missing follow-up information or survival time < 3 months (*n* = 460). The final analytic cohort included 24,215 surgically treated stage IA NSCLC patients (Fig. 1).

### Exposure and covariates

The primary exposure was the extent of nodal evaluation, quantified by examined lymph nodes (ELN) from the “Regional nodes examined” variable. The primary analysis compared ELN < 6 versus ELN ≥ 6. We prespecified ELN ≥ 6 as the threshold for “adequate” nodal evaluation, in line with international staging recommendations (AJCC/UICC/IASLC/ESTS) that generally require removal of at least six regional lymph nodes for a reliable pN0 designation and with contemporary data from ACOSOG Z0030 showing that complete systematic mediastinal dissection almost invariably yields ≥ 6 nodes [16–18]. In sensitivity analyses, we applied a more stringent definition of adequacy using ELN ≥ 10 (ELN < 10 vs. ELN ≥ 10), reflecting the historical American College of Surgeons Commission on Cancer “10 regional lymph nodes” (10RLN) quality metric for resected stage I–II NSCLC and real-world evidence that examining ≥ 10 nodes is associated with more accurate staging and better survival in N0 disease [12, 19]. To avoid misclassification of the reference group, we restricted the cohort to patients with regional nodes examined ≥ 1 rather than including patients with no nodal evaluation.

Baseline covariates were defined from SEER at diagnosis and included demographic, tumor, treatment, and socioeconomic factors. Demographic variables were age (< 60 vs. ≥ 60 years), sex, race (White vs. non-White), and marital status (married vs. unmarried; unmarried included divorced, widowed, and never married). Tumor characteristics included grade (I, II, III–IV), tumor size (≤ 1 cm, 1–2 cm, 2–3 cm), primary site (upper lobe, lower lobe, others including middle lobe and overlapping/not otherwise specified), laterality (left vs. right), and histology (adenocarcinoma [AC] vs. squamous cell carcinoma [SCC]). Surgical procedure was categorized as lobectomy versus sublobar resection.

Socioeconomic variables included county-level median household income and county-level urban–rural residence. Median household income (USD) was reclassified as low (< 50,000), middle (50,000–99,999), and high (≥ 100,000). The SEER county urbanization variable was reclassified as urban (all metropolitan counties) versus rural (all nonmetropolitan counties, adjacent or nonadjacent to metropolitan areas). Because these covariates could influence both prognosis and the likelihood of more extensive nodal evaluation, they were incorporated into propensity score estimation and multivariable models.

### Outcomes

Traditional survival outcomes and competing-risk outcomes were evaluated. OS was defined as time from diagnosis to death from any cause, with patients censored at last follow-up if alive. CSS was defined as time from diagnosis to death attributed to lung cancer, with deaths from other causes treated as censored. For competing-risk analyses, CSD was defined as death with lung cancer recorded as the underlying cause of death in SEER, and OCD as death from any non–lung cancer cause. In the competing-risk framework, CSD was the event of interest and OCD the competing event. Given the favorable prognosis of stage IA NSCLC and the high proportion of older adults with substantial non-cancer mortality, the competing-risk analysis of CSD was prespecified as the primary endpoint, whereas CSS was used primarily for comparability with prior literature.

### Propensity score matching

Because the extent of nodal evaluation was not randomly assigned, we used PSM to reduce confounding in comparisons between ELN < 6 and ELN ≥ 6. Propensity scores were estimated using a multivariable logistic regression model with receipt of ELN ≥ 6 as the dependent variable and all baseline covariates listed above as predictors. We performed 1:1 nearest-neighbor matching without replacement using a caliper width of 0.10 on the logit of the propensity score. The matched cohort comprised 13,812 patients (6,906 per group). Covariate balance before and after matching was assessed primarily using standardized mean differences (SMD), with an absolute SMD < 0.10 indicating adequate balance; balance was visualized using a Love plot (Figure S1), and the full SMD values are provided in Supplementary Table S3. The P values shown in Table 1 were derived from chi-square tests and retained only for descriptive comparison, whereas SMDs were used as the primary criterion for evaluating post-matching balance.

**Table 1:** Baseline characteristics of stage IA NSCLC patients by examined lymph nodes (ELN < 6 vs. ELN ≥ 6) before and after PSM

Characteristics	Before PSM				After PSM			
	ALL	ELN < 6	ELN > = 6	*P* value	ALL	ELN < 6	ELN > = 6	*P* value
	*N* = 24,215	*N* = 10,229	*N* = 13,986		*N* = 13,812	*N* = 6906	*N* = 6906	
Age:				0.722				<0.001
<60	4037 (16.7%)	1716 (16.8%)	2321 (16.6%)		2173 (15.7%)	1223 (17.7%)	950 (13.8%)	
>=60	20,178 (83.3%)	8513 (83.2%)	11,665 (83.4%)		11,639 (84.3%)	5683 (82.3%)	5956 (86.2%)	
Gender:				0.834				0.016
Female	13,935 (57.5%)	5878 (57.5%)	8057 (57.6%)		8079 (58.5%)	3969 (57.5%)	4110 (59.5%)	
Male	10,280 (42.5%)	4351 (42.5%)	5929 (42.4%)		5733 (41.5%)	2937 (42.5%)	2796 (40.5%)	
Race:				0.010				<0.001
White	20,527 (84.8%)	8599 (84.1%)	11,928 (85.3%)		11,781 (85.3%)	5745 (83.2%)	6036 (87.4%)	
Non White	3688 (15.2%)	1630 (15.9%)	2058 (14.7%)		2031 (14.7%)	1161 (16.8%)	870 (12.6%)	
Marital status:				0.115				<0.001
Married	13,410 (55.4%)	5604 (54.8%)	7806 (55.8%)		7866 (57.0%)	3804 (55.1%)	4062 (58.8%)	
Unmarried	10,805 (44.6%)	4625 (45.2%)	6180 (44.2%)		5946 (43.0%)	3102 (44.9%)	2844 (41.2%)	
Grade:				0.002				<0.001
I	6348 (26.2%)	2762 (27.0%)	3586 (25.6%)		3465 (25.1%)	1860 (26.9%)	1605 (23.2%)	
II	12,282 (50.7%)	5051 (49.4%)	7231 (51.7%)		7227 (52.3%)	3463 (50.1%)	3764 (54.5%)	
III/IV	5585 (23.1%)	2416 (23.6%)	3169 (22.7%)		3120 (22.6%)	1583 (22.9%)	1537 (22.3%)	
Primary site:				<0.001				<0.001
Upper lobe	14,787 (61.1%)	5997 (58.6%)	8790 (62.8%)		8490 (61.5%)	3944 (57.1%)	4546 (65.8%)	
Lower lobe	7941 (32.8%)	3477 (34.0%)	4464 (31.9%)		4236 (30.7%)	2364 (34.2%)	1872 (27.1%)	
Others	1487 (6.1%)	755 (7.4%)	732 (5.2%)		1086 (7.9%)	598 (8.7%)	488 (7.1%)	
Tumor size:				<0.001				<0.001
<=1 cm	3485 (14.4%)	1838 (18.0%)	1647 (11.8%)		1842 (13.3%)	979 (14.2%)	863 (12.5%)	
>1.0–2.0 cm	12,846 (53.0%)	5574 (54.5%)	7272 (52.0%)		6933 (50.2%)	3746 (54.2%)	3187 (46.1%)	
>2.0–3.0 cm	7884 (32.6%)	2817 (27.5%)	5067 (36.2%)		5037 (36.5%)	2181 (31.6%)	2856 (41.4%)	
Laterality:				0.527				<0.001
Left	9856 (40.7%)	4139 (40.5%)	5717 (40.9%)		6018 (43.6%)	2695 (39.0%)	3323 (48.1%)	
Right	14,359 (59.3%)	6090 (59.5%)	8269 (59.1%)		7794 (56.4%)	4211 (61.0%)	3583 (51.9%)	
Pathology:				0.077				<0.001
AC	18,698 (77.2%)	7841 (76.7%)	10,857 (77.6%)		10,375 (75.1%)	5385 (78.0%)	4990 (72.3%)	
SCC	5517 (22.8%)	2388 (23.3%)	3129 (22.4%)		3437 (24.9%)	1521 (22.0%)	1916 (27.7%)	
Surgery:				0.000				1.000
Lobectomy	16,893 (69.8%)	4907 (48.0%)	11,986 (85.7%)		9812 (71.0%)	4906 (71.0%)	4906 (71.0%)	
Sublobar	7322 (30.2%)	5322 (52.0%)	2000 (14.3%)		4000 (29.0%)	2000 (29.0%)	2000 (29.0%)	
Household income:				<0.001				0.241
High	5061 (20.9%)	2241 (21.9%)	2820 (20.2%)		2722 (19.7%)	1399 (20.3%)	1323 (19.2%)	
Low	1637 (6.8%)	728 (7.1%)	909 (6.5%)		968 (7.0%)	474 (6.9%)	494 (7.2%)	
Middle	17,517 (72.3%)	7260 (71.0%)	10,257 (73.3%)		10,122 (73.3%)	5033 (72.9%)	5089 (73.7%)	
County type:				0.082				<0.001
Rural	2963 (12.2%)	1296 (12.7%)	1667 (11.9%)		1829 (13.2%)	822 (11.9%)	1007 (14.6%)	
Urban	21,252 (87.8%)	8933 (87.3%)	12,319 (88.1%)		11,983 (86.8%)	6084 (88.1%)	5899 (85.4%)	

*P* values were calculated using chi-square tests to compare baseline characteristics between ELN groups before and after PSM and are provided for descriptive purposes. Post-matching covariate balance was assessed primarily using absolute standardized mean differences rather than P values; the full SMD values are shown in Supplementary Table S3

*Abbreviations*: *NSCLC* non–small cell lung cancer, *ELN* examined lymph nodes, *PSM* propensity score matching, *AC* adenocarcinoma, *SCC* squamous cell carcinoma﻿

### Survival and competing-risk analyses

**Fig. 2:**
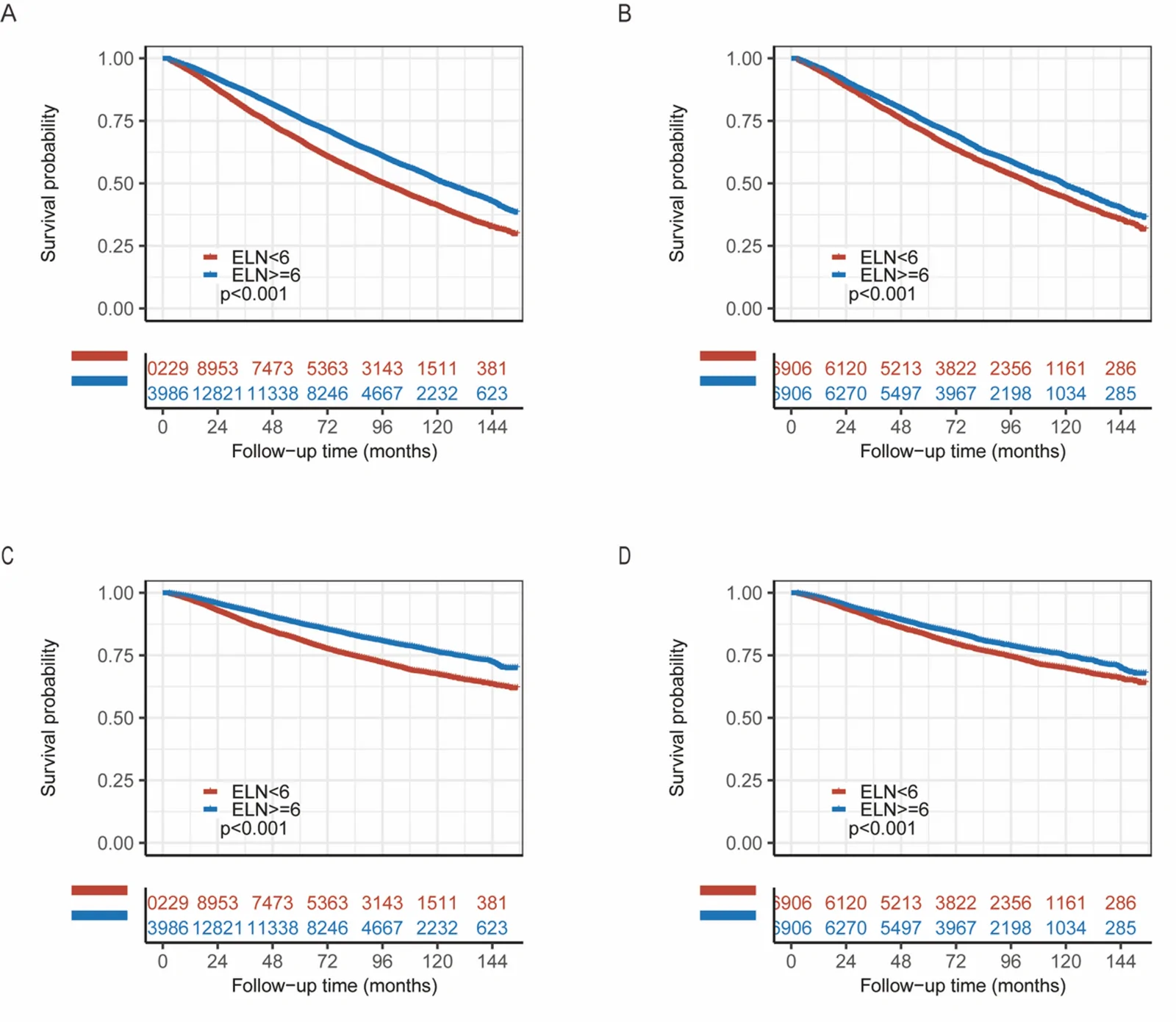
Overall and cancer-specific survival by ELN group in patients with stage IA NSCLC. **A** OS in the unmatched cohort. **B** OS in the propensity score-matched cohort. **C** CSS in the unmatched cohort. **D** CSS in the matched cohort. Survival curves were estimated using the Kaplan-Meier method and compared between ELN < 6 and ELN ≥6 using the log-rank test. Abbreviations: ELN, examined lymph nodes; OS, overall survival; CSS, cancer-specific survival; NSCLC, non-small cell lung cancer; PSM, propensity score matching

**Fig. 3:**
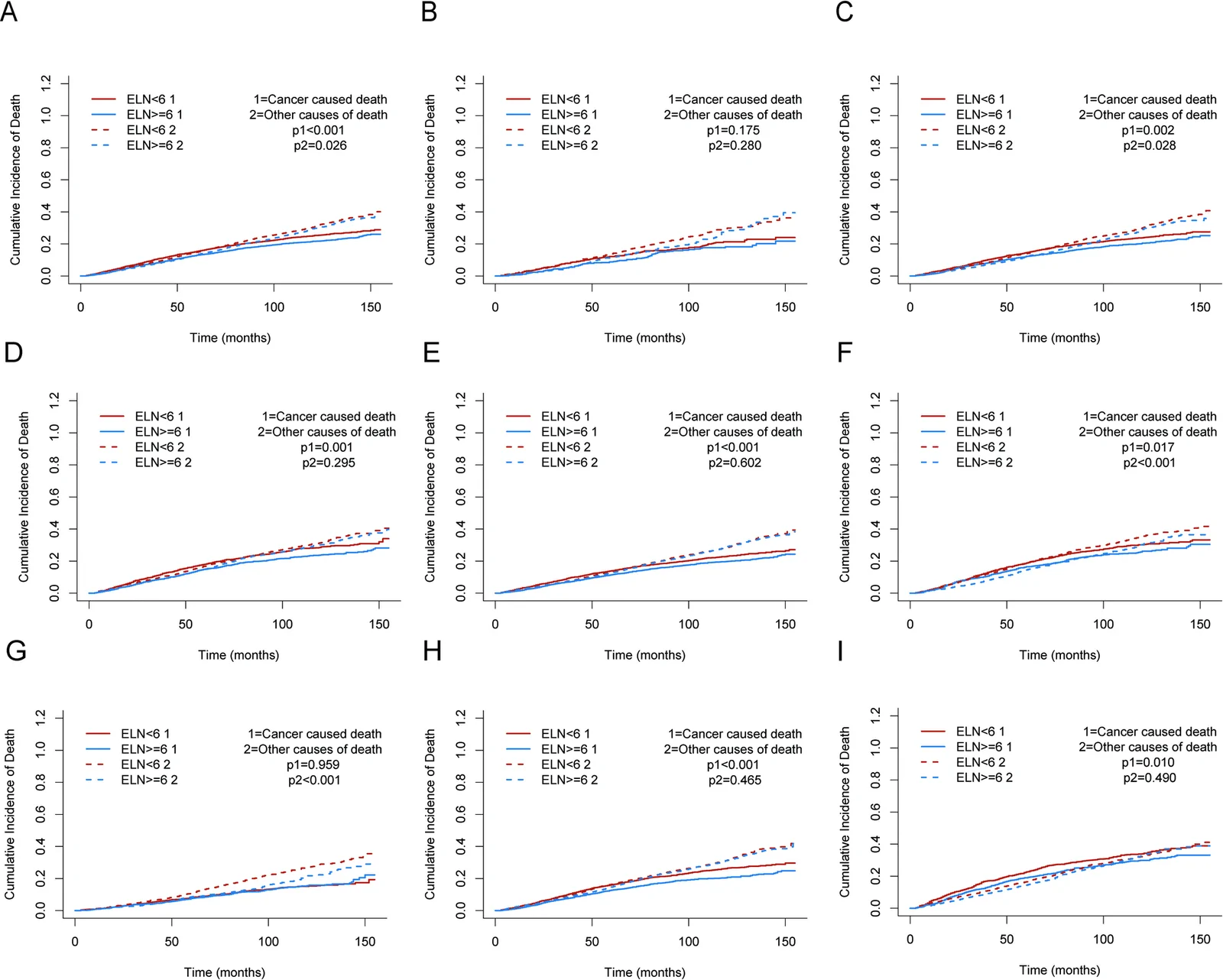
Cumulative incidence of CSD and OCD by ELN group in the PSM cohort and predefined subgroups. **A** Overall matched cohort; (**B**-**D**) tumor size ≤1 cm, 1–2 cm, and 2–3 cm, respectively; (**E**-**F**) lobectomy and sublobar resection, respectively; (**G**-**I**) Grade I, Grade II, and Grade III–IV tumors, respectively.Cumulative incidence functions were estimated within a competing-risk framework, and differences between ELN <6 and ELN ≥6 were compared using Gray’s test.Abbreviations: ELN, examined lymph nodes; CSD, cancer-specific death; OCD, other-cause death; PSM, propensity score matching; NSCLC, non–small cell lung cancer

In the unmatched and matched cohorts, OS and CSS were estimated using the Kaplan–Meier method and compared between ELN groups using the log-rank test (Fig. 2). In competing-risk analyses, cumulative incidence functions (CIFs) were used to estimate the cumulative incidence of CSD and OCD by ELN group, and Gray’s test was used to compare CIF curves (Table 2; Fig. 3).

**Table 2:** Cumulative incidence of CSD and OCD by lymph node evaluation (ELN < 6 vs. ELN ≥ 6) before and after PSM in stage IA NSCLC

	Cancer-specific death (%)			Gray’s test (χ²)	*P* value	Other causes death (%)			Gray’s test (χ²)	*P* value
	1-year CIF	3-year CIF	5-year CIF			1-year CIF	3-year CIF	5-year CIF		
Before PSM										
ELN < 6	2.80%	11.10%	17.40%	178.49	<0.001	2.50%	9.00%	15.60%	41.12	<0.001
ELN > = 6	1.60%	6.70%	11.50%			1.90%	6.60%	12.50%		
After PSM										
ELN < 6	2.70%	9.40%	15.30%	16.98	<0.001	2.40%	8.30%	14.90%	4.99	0.026
ELN > = 6	1.60%	7.60%	12.90%			2.10%	7.10%	13.20%		

*Abbreviations*: *NSCLC* non-small cell lung cancer, *CSD* cancer-specific death, *OCD* other-cause death, *ELN* examined lymph nodes, *PSM* propensity score matching, *CIF* cumulative incidence function

In the matched cohort, we fitted multivariable Fine–Gray subdistribution hazard models for CSD with OCD as the competing event. ELN group (ELN < 6 vs. ELN ≥ 6) was the primary exposure, and models adjusted for all baseline covariates. Results are reported as subdistribution hazard ratios (sHRs) with 95% confidence intervals (CIs) (Table 3).

**Table 3:** Multivariable Fine–Gray subdistribution hazard model for cancer-specific death in the propensity score–matched cohort

Characteristics	Subdistribution proportional hazards model		
	sHR	95% CI	*P* value
Age:			
<60	Reference		
>=60	1.29	1.19–1.40	< 0.001
Gender:			
Female	Reference		
Male	1.30	1.23–1.38	< 0.001
Race:			
White	Reference		
Non White	0.86	0.80–0.94	< 0.001
Marital status:			
Married	Reference		
Unmarried	1.18	1.11–1.25	< 0.001
Grade:			
I	Reference		
II	1.61	1.49–1.74	< 0.001
III/IV	2.12	1.94–2.32	< 0.001
Primary site:			
Upper lobe	Reference		
Lower lobe	1.06	1.00-1.13	0.051
Others	1.13	1.01–1.27	0.040
Tumor size:			
<=1 cm	Reference		
>1.0–2.0 cm	1.09	1.00-1.19	0.058
>2.0–3.0 cm	1.30	1.19–1.43	< 0.001
Laterality:			
Left	Reference		
Right	1.04	0.98–1.10	0.210
Pathology:			
AC	Reference		
SCC	1.08	1.01–1.15	0.023
Surgery:			
Lobectomy	Reference		
Sublobar	1.50	1.40–1.60	< 0.001
Household income:			
High	Reference		
Low	1.29	1.12–1.48	< 0.001
Middle	1.15	1.07–1.24	< 0.001
County type:			
Rural	Reference		
Urban	0.82	0.75–0.91	< 0.001
group:			
ELN < 6	Reference		
ELN > = 6	0.78	0.74–0.83	< 0.001

*Abbreviations*: *sHR* subdistribution hazard ratio, *CI* confidence interval, *CSD* cancer-specific death, *ELN* examined lymph nodes, *AC *adenocarcinoma, *SCC *squamous cell carcinoma

### Subgroup and sensitivity analyses

Prespecified subgroup analyses were conducted in the matched cohort to examine whether associations differed across clinically relevant strata, including tumor size, grade, surgical procedure, age, sex, race, primary site, marital status, income, residence (urban vs. rural), laterality, and histology. Within each subgroup, CIFs for CSD and OCD were compared between ELN < 6 and ELN ≥ 6 using Gray’s test (Fig. 3 B and I).

For sensitivity analyses, we redefined “adequate” nodal evaluation using ELN ≥ 10 and repeated all analyses comparing ELN < 10 versus ELN ≥ 10 in unmatched and matched cohorts, including Kaplan–Meier analyses for OS/CSS, CIF/Gray’s tests for CSD/OCD, and multivariable Fine–Gray models in the matched cohort (Figures S2–S3; Tables S1–S2).

### Statistical analysis

All analyses were performed using R software (version 4.2.1). Propensity score estimation and matching used the MatchIt package; balance assessment and Love plots used cobalt; Kaplan–Meier analyses used survival and survminer; and competing-risk analyses (CIF, Gray’s test, and Fine–Gray regression) used cmprsk. All tests were two-sided, with *P* < 0.05 considered statistically significant.

## Results

### Baseline characteristics and propensity score matching

A total of 24,215 surgically treated stage IA NSCLC patients were included, of whom 10,229 (42.3%) had ELN < 6 and 13,986 (57.7%) had ELN ≥ 6 (Table 1). The cohort was predominantly older (≥ 60 years, 83.3%), and most patients were White (84.8%), had adenocarcinoma (77.2%), and were married. Tumors most commonly arose in the upper lobe, and tumor size was 1–2 cm in 53.0% and 2–3 cm in 32.6%. Lobectomy was performed in 69.8% of patients, with the remaining patients undergoing sublobar resection. Most patients resided in urban areas (87.8%), and the majority had middle-level county median household income (72.3%).

Before matching, compared with the ELN < 6 group, patients with ELN ≥ 6 were more likely to be White, married, have higher tumor grade, upper-lobe primaries, and tumors measuring 1–3 cm; they were also more likely to undergo lobectomy and to live in urban and middle-to-high income counties. Conversely, sublobar resection, low income, and rural residence were more common in the ELN < 6 group. These differences were most pronounced for race, tumor grade and size, primary site, surgical procedure, and household income (all *P* < 0.05), whereas age, sex, marital status, histology, laterality, and residence type were broadly similar between groups (all *P* > 0.05).

After incorporating the clinical and socioeconomic covariates into the propensity score model, 1:1 nearest-neighbor matching yielded 13,812 patients (6,906 per group). Post-matching, covariate balance was assessed primarily using standardized mean differences rather than P values. All absolute SMDs were < 0.10, and the full SMD values are provided in Supplementary Table S3, while covariate balance is visualized in Figure S1, indicating acceptable balance for subsequent survival and competing-risk analyses.

### Overall survival and cancer-specific survival by ELN group

Kaplan–Meier analyses demonstrated that patients with ELN ≥ 6 had significantly better OS and CSS than those with ELN < 6 (Fig. 2). The median follow-up for the overall cohort was 102 months. In the unmatched cohort, OS and CSS curves separated early after surgery and remained separated throughout follow-up (Fig. 2A and C; log-rank *P* < 0.001 for both). After PSM, the same pattern persisted: ELN ≥ 6 remained associated with superior OS and CSS (Fig. 2B and D; log-rank *P* < 0.001 for both). Although the absolute separation between curves was modestly attenuated after matching, the consistently higher survival probability in the ELN ≥ 6 group suggests that the association between more extensive nodal evaluation and improved survival was not solely driven by baseline imbalance.

### Competing-risk analyses of cancer-specific death and other-cause death

Given the high frequency of competing deaths in this predominantly older cohort, we evaluated CSD and OCD using competing-risk methods (Table 2; Fig. 3). In the unmatched cohort, the 1-, 3-, and 5-year CIFs for CSD were 2.80%, 11.10%, and 17.40% for ELN < 6 versus 1.60%, 6.70%, and 11.50% for ELN ≥ 6 (Gray’s χ²=178.49, *P* < 0.001). OCD showed a similar pattern (2.50%, 9.00%, and 15.60% vs. 1.90%, 6.60%, and 12.50%; χ²=41.12, *P* < 0.001). Thus, before accounting for baseline differences, more extensive nodal evaluation was associated with lower risks of both CSD and OCD.

After matching, the advantage of ELN ≥ 6 for CSD remained statistically significant. The 1-, 3-, and 5-year CIFs for CSD were 2.70%, 9.40%, and 15.30% for ELN < 6 versus 1.60%, 7.60%, and 12.90% for ELN ≥ 6 (χ²=16.98, *P* < 0.001). This corresponded to an absolute risk reduction (ARR) of 2.4% for 5-year CSD, with the absolute separation increasing over time (ARR 1.1%, 1.8%, and 2.4% at 1, 3, and 5 years, respectively). Although this difference was statistically significant, the absolute magnitude of effect remained modest. For OCD, CIFs were 2.40%, 8.30%, and 14.90% versus 2.10%, 7.10%, and 13.20% (χ²=4.99, *P* = 0.026), with smaller absolute differences. As shown in Fig. 3A, CSD curves diverged early and progressively over time, whereas OCD curves separated only slightly, suggesting that the observed association was more evident for cancer-related mortality than for non-cancer mortality.

Prespecified subgroup competing-risk analyses in the matched cohort showed broadly consistent associations between ELN ≥ 6 and lower CSD across most clinical strata (Fig. 3B and I). By tumor size, CIF curves for both CSD and OCD were nearly overlapping in tumors ≤ 1 cm (Fig. 3B), suggesting limited marginal benefit from more extensive nodal evaluation in ultra-small lesions. In contrast, for tumors 1–2 cm and 2–3 cm, the CSD CIF for ELN ≥ 6 was consistently lower than for ELN < 6 from early follow-up onward, with relatively small differences in OCD (Fig. 3C and D), supporting a more evident association with lower cancer-specific mortality when tumor size exceeded 1 cm. By surgical procedure, ELN ≥ 6 was associated with lower CSD CIF after both lobectomy and sublobar resection; OCD was largely unchanged after lobectomy and slightly lower after sublobar resection (Fig. 3E and F). By grade, ELN ≥ 6 was associated with persistently lower CSD CIF in Grade II and Grade III–IV tumors, whereas CSD curves were nearly overlapping in Grade I tumors, with only minor differences in OCD (Fig. 3G and I). By age, no significant differences in CSD or OCD were observed between ELN groups among patients younger than 60 years (Gray’s *P* = 0.548 and 0.829, respectively), whereas among those aged ≥ 60 years, ELN ≥ 6 was associated with significantly lower CSD and OCD (Gray’s *P* < 0.001 and 0.002, respectively). Across other subgroups defined by sex, race, primary site, marital status, income, residence, laterality, and histology, ELN ≥ 6 was generally associated with lower or at least not higher CSD, while differences in OCD were small and no reversal of effect direction was observed.

### Multivariable Fine–Gray model for cancer-specific death

In the matched cohort, multivariable Fine–Gray regression identified independent predictors of CSD (Table 3). Older age, male sex, unmarried status, higher grade, larger tumor size, SCC histology, sublobar resection, and lower household income were associated with increased CSD risk, whereas urban residence and ELN ≥ 6 were associated with lower CSD risk. Compared with age < 60 years, age ≥ 60 years was associated with increased CSD. Male sex, unmarried status, and low-to-middle income were also associated with higher risk relative to their respective reference groups. Relative to Grade I and tumor size ≤ 1 cm, higher-grade tumors and tumors 2–3 cm were associated with substantially higher CSD risk; SCC had a modestly increased risk compared with AC.

Among treatment-related factors, sublobar resection was associated with the largest increase in CSD risk compared with lobectomy (sHR = 1.50, 95% CI 1.40–1.60; *P* < 0.001). Importantly, after adjustment for all clinical and socioeconomic covariates, ELN ≥ 6 remained independently associated with an approximately 22% lower CSD risk compared with ELN < 6 (sHR = 0.78, 95% CI 0.74–0.83; *P* < 0.001).

### Sensitivity analyses using ELN ≥ 10

Sensitivity analyses redefining adequate nodal evaluation as ELN ≥ 10 are shown in Figures S2–S3 and Tables S1–S2. In both unmatched and matched cohorts, ELN ≥ 10 was associated with consistently higher OS and CSS than ELN < 10 (Figure S2; log-rank *P* < 0.001 for all). In competing-risk analyses, ELN ≥ 10 was also associated with lower CIFs for both CSD and OCD: before matching, 5-year CSD decreased from 15.6% to 11.3% and 5-year OCD from 15.0% to 11.9%; after matching, 5-year CSD decreased from 14.1% to 11.3% and 5-year OCD from 14.4% to 11.8% (all Gray’s tests *P* < 0.001; Figure S3, Table S1). In multivariable Fine–Gray models, ELN ≥ 10 remained significantly associated with lower CSD risk (sHR = 0.85, 95% CI 0.80–0.91; *P* < 0.001; Table S2). Compared with the primary analysis using ELN ≥ 6, the association observed with ELN ≥ 10 was slightly attenuated but directionally consistent, supporting the robustness of the core finding that inadequate nodal evaluation is an adverse prognostic factor in stage IA NSCLC.

## Discussion

In this large SEER-based cohort study, we focused on surgically treated pathologic stage IA (T1N0M0) NSCLC and evaluated whether “more thorough” lymph node (LN) evaluation—at levels recommended by existing staging systems and quality metrics—was associated with more favorable oncologic outcomes in a real-world IA N0 population [20, 21]. We found that, when using ELN ≥ 6 as the primary threshold, the 5-year cancer-specific death decreased from 15.3% to 12.9% after matching, corresponding to a subdistribution hazard ratio of approximately 0.78. Although the absolute risk reduction was modest (ARR 2.4% at 5 years), this difference may still be clinically meaningful in a generally favorable-prognosis IA cohort. At the same time, differences in other-cause death were relatively small, although lower OCD was also observed in some comparisons among patients with more extensive lymph node evaluation. This finding may reflect residual confounding related to patient selection, baseline health status, comorbidity burden, surgical expertise, and perioperative care quality, rather than a direct protective effect of lymph node evaluation on non-cancer mortality. Therefore, the observed survival differences should still be interpreted as associative and hypothesis-generating rather than causal [22]. Sensitivity analyses using ELN ≥ 10 as a more stringent reference threshold showed a directionally consistent association with slightly attenuated effect size, further reinforcing the robustness of the association between inadequate LN evaluation and worse prognosis [23, 24].

Compared with previous work, our study offers a more focused analysis of this question. First, multiple SEER-based and multicenter registry studies have reported that examining a greater number of LNs is associated with more accurate staging and improved survival, but most of these cohorts combined stage I–III disease and multiple surgical procedures, used OS or CSS as endpoints, and rarely applied competing-risk methods in truly early-stage, pathologic N0 settings. In contrast, we strictly restricted the cohort to pathologic T1N0M0 disease, included only lobectomy and sublobar resection, and used cancer-specific death with Fine–Gray competing-risk models, thereby providing a more focused evaluation in a pathologic IA cohort. Second, our aim was not to “redefine” an optimal LN count cutoff within the IA population, but rather to preselect 6 and 10 nodes as reference thresholds based on staging consensus and quality metrics, and then examine whether, in an IA N0 cohort, reaching these established levels was associated with lower cancer-specific mortality, and whether this association was uniform across all patients. Our results indicate that intensified LN evaluation is overall associated with lower cancer-specific mortality, but this association clearly depended on tumor size: when tumor diameter exceeds 1 cm, the association between ELN ≥ 6 and lower cancer-specific mortality becomes more apparent, whereas in ≤ 1 cm ultra-low-risk lesions, the cumulative incidence curves for cancer-specific death are nearly superimposed and the marginal effect is limited [23, 25–27].

This size-dependent pattern of association is biologically and staging-wise plausible. Numerous studies have demonstrated that the rate of occult LN metastasis in T1 tumors increases significantly with tumor diameter, and that inadequate LN evaluation is more likely to result in N1/N2 understaging and undertreatment [28–30]. For tumors measuring 1–3 cm, examining at least six LNs may increase the likelihood of detecting occult nodal disease and, by triggering closer surveillance or adjuvant therapy, can translate into a reduction in cancer-specific mortality. In contrast, for subcentimeter lesions with an intrinsically low baseline risk of nodal involvement—especially in older patients with substantial comorbidity—the additional benefit of removing a few more nodes may be limited relative to the procedural complexity and potential risk [31]. Taken together, our findings suggest that the association between intensified LN evaluation and lower cancer-specific mortality was more apparent in tumors > 1 cm, whereas this association appeared limited in tumors ≤ 1 cm.

Against the backdrop of a growing emphasis on parenchymal-sparing surgery, our study also provides complementary insights into the relationship between surgical procedure and LN evaluation [2]. In the matched cohort, regardless of whether patients underwent lobectomy or sublobar resection, the cumulative incidence of cancer-specific death was lower in the ELN ≥ 6 group than in the ELN < 6 group, while differences in other-cause death remained small. This suggests that, at the reference thresholds used, more thorough LN evaluation was associated with lower cancer-specific mortality across both procedures. At the same time, sublobar resection remained an independent adverse prognostic factor in the multivariable competing-risk model, which is consistent with current guideline recommendations that segmentectomy or wedge resection must be accompanied by systematic hilar and mediastinal LN evaluation to avoid understaging and undertreatment. In other words, adequate LN evaluation should be regarded as a basic requirement for both lobectomy and sublobar resection. When considering parenchymal-sparing surgery, the ability—when anatomically feasible—to achieve an LN evaluation level comparable to ELN ≥ 6 and preferably approaching ELN ≥ 10 should be viewed as an important component of oncologic safety rather than a detail that can be overlooked [32].

This study has several strengths. We constructed a large, pathologically homogeneous IA N0 cohort with clearly defined surgical procedures, incorporated multidimensional covariates including socioeconomic factors into the analytic framework, and used competing-risk models to distinguish tumor-related from non–tumor-related mortality, allowing a clearer view of the prognostic information carried by LN evaluation itself [33]. Nonetheless, several limitations must be acknowledged. First, as a retrospective SEER-based analysis, we lacked information on comorbidities, pulmonary function, performance status, specific nodal stations, and intraoperative details, all of which may influence both the extent of LN evaluation and long-term outcomes; residual confounding cannot be fully excluded. In particular, SEER does not capture hospital volume, surgeon specialization, or the intensity of multidisciplinary oncologic management, all of which may have influenced both nodal evaluation and prognosis. Second, the “Regional nodes examined” variable records only the total number of nodes removed and does not reflect the specific nodal stations evaluated, mediastinal versus hilar assessment, or systematic sampling versus formal dissection; therefore, ELN serves only as an approximate surrogate for the quality of nodal evaluation rather than a comprehensive measure of nodal assessment [34]. Third, misclassification of cause of death is possible in registry data [35]. Finally, our findings reflect practice patterns in the United States, and caution is needed when extrapolating to regions with different lung cancer epidemiology, surgical standards, and healthcare resources.

Despite these limitations, our study provides several relatively clear and clinically relevant messages for LN evaluation in contemporary IA NSCLC. First, at LN evaluation levels consistent with current staging systems and quality metrics, achieving a “relatively thorough” assessment—such as ELN ≥ 6, with ELN ≥ 10 as a reference—was overall associated with lower cancer-specific mortality in IA N0 patients. Second, this association is not uniform across all patients but is mainly concentrated among those with tumors > 1 cm in diameter. Third, for tumors ≤ 1 cm, particularly in older or highly comorbid patients, the apparent association of more extensive nodal evaluation may be limited; however, this finding should be interpreted cautiously and should not be considered a direct recommendation for de-escalation without prospective validation. Future prospective cohorts and high-quality registry studies are needed to more finely characterize the balance between LN number, nodal station coverage, perioperative risk, and patient-reported outcomes, and to further refine lymph node evaluation strategies based on tumor size and overall risk profile, with the aim of better balancing oncologic adequacy against surgical cost in this relatively favorable-prognosis population of IA NSCLC.

## Supplementary Information


Supplementary Material 1.


## Data Availability

The datasets used and/or analyzed during the current study are available from the Surveillance, Epidemiology, and End Results (SEER) Program of the US National Cancer Institute (https://seer.cancer.gov). The SEER data are de-identified and publicly accessible after registration and submission of a data-use agreement. Additional analytic code or materials are available from the corresponding author on reasonable request.
