# Supplementary material for: Risk-adapted nodal evaluation in stage IA non–small cell lung cancer: a population-based competing-risk analysis
Source: World J Surg Oncol. 2026 Jul 1;24:352. doi: 10.1186/s12957-026-04485-2 (PMC13525672; doi:10.1186/s12957-026-04485-2)
Supplement: Supplementary file 1 [file 12957_2026_4485_MOESM1_ESM.docx]

**Figure S1. Covariate balance between ELN <6 and ELN ≥6 before and after propensity score matching.**
Love plot showing absolute standardized mean differences for baseline demographic, tumor, treatment, and socioeconomic variables in the unmatched and matched cohorts. The dashed vertical line denotes an absolute standardized mean difference of 0.10, prespecified as the threshold for acceptable balance between groups.
**Abbreviations:** ELN, examined lymph nodes; PSM, propensity score matching.

**Figure S2. Overall and cancer-specific survival by ELN group using a threshold of 10 nodes.**
Kaplan–Meier curves comparing patients with ELN <10 versus ELN ≥10.
(A) OS in the unmatched cohort.
(B) OS in the PSM cohort.
(C) CSS in the unmatched cohort.
(D) CSS in the PSM cohort.
**Abbreviations:** ELN, examined lymph nodes; OS, overall survival; CSS, cancer-specific survival; PSM, propensity score matching.

**Figure S3. Cumulative incidence of CSD and OCD by ELN group using a threshold of 10 nodes in the PSM cohort.**
CIF curves compare ELN <10 versus ELN ≥10 for CSD and OCD.
(A) Overall cohort.
(B) Tumor size ≤1 cm.
(C) Tumor size 1–2 cm.
(D) Tumor size 2–3 cm.
(E) Lobectomy.
(F) Sublobar resection.
(G) Grade I tumors.
(H) Grade II tumors.
(I) Grade III–IV tumors.
**Abbreviations:** ELN, examined lymph nodes; CSD, cancer-specific death; OCD, other-cause death; CIF, cumulative incidence function; PSM, propensity score matching.

Table S1 Cumulative incidence of CSD and OCD by lymph node evaluation (ELN <10 vs ELN ≥10) before and after PSM in stage IA NSCLC

|  | Cancer-specific death (%) | | | Gray’s test (χ²) | P value | Other causes death (%) | | | Gray’s test (χ²) | P value |
| --- | --- | --- | --- | --- | --- | --- | --- | --- | --- | --- |
|  | 1-year CIF | 3-year CIF | 5-year CIF |  |  | 1-year CIF | 3-year CIF | 5-year CIF |  |  |
| Before PSM |  |  |  |  |  |  |  |  |  |  |
| ELN<10 | 2.40% | 9.70% | 15.60% | 79.26 | ＜0.001 | 2.30% | 8.40% | 15.00% | 30.72 | ＜0.001 |
| ELN>=10 | 1.50% | 6.60% | 11.30% |  |  | 1.80% | 6.30% | 11.90% |  |  |
| After PSM |  |  |  |  |  |  |  |  |  |  |
| ELN<10 | 2.50% | 8.70% | 14.10% | 16.24 | ＜0.001 | 2.20% | 7.70% | 14.40% | 16.03 | ＜0.001 |
| ELN>=10 | 1.50% | 6.50% | 11.30% |  |  | 1.70% | 6.20% | 11.80% |  |  |

Abbreviations: NSCLC, non-small cell lung cancer; CSD, cancer-specific death; OCD, other-cause death; ELN, examined lymph nodes; PSM, propensity score matching;

| Table S2 The cumulative incidences and multivariate subdistribution proportional hazards analysis on CSD | | | |
| --- | --- | --- | --- |
| Characteristics | Subdistribution proportion hazards model | | |
|  | sHR | 95% CI | P value |
| Age: |  |  |  |
| <60 | Reference |  |  |
| >=60 | 1.29 | 1.19-1.40 | <0.001 |
| Gender: |  |  |  |
| Female | Reference |  |  |
| Male | 1.31 | 1.23-1.38 | <0.001 |
| Race: |  |  |  |
| White | Reference |  |  |
| Non White | 0.87 | 0.80-0.94 | <0.001 |
| Marital status: |  |  |  |
| Married | Reference |  |  |
| Unmarried | 1.18 | 1.11-1.25 | <0.001 |
| Grade: |  |  |  |
| I | Reference |  |  |
| II | 1.61 | 1.49-1.74 | <0.001 |
| III/IV | 2.12 | 1.94-2.32 | <0.001 |
| Primary site: |  |  |  |
| Upper lobe | Reference |  |  |
| Lower lobe | 1.06 | 1.00-1.13 | 0.044 |
| Others | 1.15 | 1.03-1.30 | 0.016 |
| Tumor size: |  |  |  |
| <=1cm | Reference |  |  |
| >1.0–2.0cm | 1.08 | 0.99-1.18 | 0.075 |
| >2.0–3.0cm | 1.29 | 1.18-1.42 | <0.001 |
| Laterality: |  |  |  |
| Left | Reference |  |  |
| Right | 1.04 | 0.98-1.10 | 0.160 |
| Pathology: |  |  |  |
| AC | Reference |  |  |
| SCC | 1.08 | 1.01-1.16 | 0.021 |
| Surgery: |  |  |  |
| Lobectomy | Reference |  |  |
| Sublobar | 1.59 | 1.50-1.69 | <0.001 |
| Household income: |  |  |  |
| High | Reference |  |  |
| Low | 1.3 | 1.13-1.49 | <0.001 |
| Middle | 1.15 | 1.07-1.24 | <0.001 |
| County type: |  |  |  |
| Rural | Reference |  |  |
| Urban | 0.83 | 0.75-0.91 | <0.001 |
| group: |  |  |  |
| ELN<10 | Reference |  |  |
| ELN>=10 | 0.85 | 0.80-0.91 | <0.001 |

sHR: subdistribution hazard ratio; CIF, cumulative incidences function; CSD, cancer specific death

| Table S3. Absolute standardized mean differences of baseline covariates before and after propensity score matching | | |
| --- | --- | --- |
| **Characteristics** | **Absolute SMD Before matching** | **Absolute SMD After matching** |
| Age (≥60 years) | 0.002 | 0.039 |
| Gender (Male) | 0.001 | 0.020 |
| Race (Non White) | 0.012 | 0.042 |
| Marital status (Unmarried) | 0.010 | 0.037 |
| Grade (I) | 0.014 | 0.036 |
| Grade (II) | 0.023 | 0.043 |
| Grade (III/IV) | 0.014 | 0.006 |
| Primary site (Upper lobe) | 0.042 | 0.086 |
| Primary site (Lower lobe) | 0.020 | 0.071 |
| Primary site (Others) | 0.024 | 0.015 |
| Tumor size (≤1 cm) | 0.061 | 0.017 |
| Tumor size (>1.0–2.0 cm) | 0.025 | 0.081 |
| Tumor size (>2.0–3.0 cm) | 0.085 | 0.097 |
| Laterality (Right) | 0.003 | 0.091 |
| Pathology (SCC) | 0.010 | 0.057 |
| Surgery (Sublobar resection) | 0.376 | 0.000 |
| Household income (High) | 0.020 | 0.010 |
| Household income (Low) | 0.011 | 0.003 |
| Household income (Middle) | 0.024 | 0.008 |
| County type (Urban) | 0.008 | 0.027 |
